# Vegetable–Mushroom Rotation Increases Morel (*Morchella esculenta* L.) Yields by Improving Soil Micro-Environments and Enhancing Overall Soil Quality

**DOI:** 10.3390/plants14213317

**Published:** 2025-10-30

**Authors:** Lijuan Zhang, Baohua Si, Minghao Lv, Qiannan Zhu, Han Du, Wenshu Ma, Jisong Qu

**Affiliations:** Institute of Horticulture, Ningxia Academy of Agricultural and Forestry Sciences, Yinchuan 750002, China; juanzi800219@163.com (L.Z.); 15769589562@163.com (B.S.); 18408660467@163.com (M.L.); ne-feli@126.com (Q.Z.); 18895005990@163.com (H.D.); mws9959@163.com (W.M.)

**Keywords:** *Morchella esculenta*, crop rotation, soil physicochemical properties, heavy metals, soil enzymes, allelochemicals

## Abstract

Continuous cropping of morel represents a crucial bottleneck that restricts the sustainable development of its industry. To explore the effects and mechanisms of crop rotations in alleviating continuous cropping obstacles, field experiments were conducted over two cropping years. With morel monoculture serving as the control (Control), four rotation patterns were established: tomato–morel (TM), pepper–morel (PM), watermelon–morel (WM), and cabbage–morel (CM). Soil physical and chemical properties, enzyme activities, phenolic acid substances, amino sugars, toxic metal contents, and morel yields were systematically measured. The soil quality index area (SQI-area) was employed for comprehensive evaluation. The results indicated that, in comparison to the control, rotation effectively mitigated soil salinization, optimized nutrient availability, and significantly decreased the accumulation of multiple auto-toxic phenolic acids (such as phthalic acid and benzoic acid) and toxic metals (As, Cd). All rotation treatments significantly enhanced the overall soil quality. Compared to the control, the SQI-area in rotation treatments increased by 25–137% in 2024 and 126–276% in 2025. Among these, the PM treatment exhibited the greatest increase. Furthermore, in both 2024 and 2025, the PM treatment exhibited the most substantial increase in yield. Specifically, it showed increases of 76% and 241% when compared to the control. In summary, crop rotations, particularly the pepper–morel rotation pattern, can effectively mitigate continuous cropping obstacles via multiple soil improvement mechanisms. This makes it an effective strategy for facilitating the sustainable production of morel.

## 1. Introduction

Morel *(Morchella esculenta* L.) is an edible and medicinal fungus that is widely distributed across the globe. It is rich in 17 essential amino acids, organic germanium, and polysaccharides [1]. Moreover, it exhibits anti-tumor, immune-regulating, and antioxidant properties, thus being regarded as a high-quality nutritional supplement for the human body [2]. Nevertheless, due to habitat destruction and over-harvesting, the wild resources of morels are becoming increasingly scarce. In recent years, with the breakthrough and popularization of facility cultivation techniques, the scale of artificial cultivation of morel has gradually expanded. China is among the earliest countries to attempt the artificial cultivation of morel, with such attempts dating back to the mid- and late 20th century [3,4]. Currently, the actual annual cultivation area of morel in China remains stable at 16,000–20,000 ha [5].

Although remarkable progress has been achieved in the artificial cultivation of morel, continuous cropping obstacles have become a crucial bottleneck restricting the sustainable development of this industry. Practical experience has shown that continuous cultivation of morel often results in weak mycelial growth, a higher incidence of malformed fruiting bodies, and frequent diseases. Ultimately, this leads to a significant decline in yield and seriously impacts the economic benefits of growers. Existing research has verified that the continuous cropping obstacles of morel result from the combined action of multiple factors. Firstly, continuous cropping can cause the deterioration of soil physical and chemical properties. This is manifested as soil compaction, pH acidification or alkalization (depending on the initial properties of the soils), and secondary salinization, which disrupts the suitable microenvironment for the growth of morel [6,7,8]. Secondly, the accumulation of allelochemicals such as organic acids and phenols secreted by morel itself, along with the release of amine and aldehyde toxins from the decomposition of previous crop residues in the soil, leads to autotoxicity when morel is replanted and aggravates the continuous cropping obstacles [9,10]. Additionally, continuous cropping disturbs the balance of soil microbial communities, leading to the enrichment of pathogenic or harmful saprophytic fungi. These not only directly cause diseases but also inhibit the growth of morel mycelia by competing for nutrients and space [11,12]. Thus, exploring a green solution to the continuous cropping obstacles that can balance yield benefits and ecological sustainability has become an urgent requirement for the current development of the morel industry.

Crop rotation is a traditional approach employed to mitigate continuous cropping issues in agricultural production. Its fundamental mechanism involves altering the types of preceding crops, thereby attaining multiple soil improvement benefits. These include enhancing soil physical and chemical properties, regulating soil microbial characteristics, and reducing the accumulation of allelochemicals [13,14]. Ultimately, these effects enhance soil quality and establish favorable soil conditions for subsequent crops. Currently, crop rotation has been extensively utilized and proven effective in the production of vegetables, grains, and certain edible fungi (such as *Lentinula edodes* and *Pleurotus ostreatus*) [6,13,14,15]. However, in the specific context of morel cultivation, the selection of appropriate rotation crops and the systematic exploration of the impacts of different rotation patterns on morel yields, soil physical and chemical indicators, soil enzyme activity, and allelochemical content remain relatively limited. Simultaneously, the mechanism by which crop rotation alleviates the continuous cropping obstacles of morel is not sufficiently clear, making it challenging to provide a scientific foundation for the sustainable cultivation of morel.

Notably, the effectiveness of crop rotation in improving soil environments is closely associated with the exudate characteristics of preceding crops, especially the fundamental differences between mushroom and plant exudates. Unlike plant root exudates, which are mainly composed of low-molecular-weight organic acids, sugars, and amino acids (primarily functioning in activating mineral nutrients and recruiting rhizosphere microorganisms), as well as proteins and large structures vesicles, mushroom exudates (released by mycelia during growth) and decomposed spent mushroom substrate contain different components such as chitin, glucans, and lignocellulolytic enzymes (e.g., cellulase, laccase) [6,7,8,9,10]. These mushroom-derived substances may exert autotoxic effects on mushrooms themselves, but they also offer beneficial functions (e.g., accelerating the decomposition of recalcitrant soil organic matter, providing specific carbon sources, and promoting the proliferation of soil microorganisms). This unique advantage of mushroom exudates suggests that vegetable–mushroom rotation may exert a more targeted regulatory effect on the soil microecosystem. Compared with traditional crop rotation systems, it may more effectively alleviate the continuous cropping obstacles of morels.

In light of this, a field experiment was carried out in the main production area of morel in Ningxia, China, from 2023 to 2025. The experiment designated morel monoculture as the control (morel–morel), and simultaneously established four rotation treatments, including tomato–morel, pepper–morel, watermelon–morel, and cabbage–morel. The rotation system included one season of rotational crops (no morel cultivation) followed by one season of morel cultivation, which allowed us to evaluate the impact of ‘temporary absence of morels’ on soil properties and subsequent morel yields. During the experiment, the yield of morel under each treatment was measured, along with soil chemical properties, enzymes, heavy metals, allelochemicals (phenolic acids), and microbial source indicators (amino sugars). Notably, soil amino sugars, as key microbial source indicators, play an irreplaceable role in exploring the mechanism of crop rotation regulating soil microecosystems. They are specific components of microbial cell walls (e.g., glucosamine from fungi and muramic acid from bacteria) and can stably exist in soil. This was to comprehensively explore the impact of crop rotation on the soil micro-ecosystem. The aims of this study were (i) to screen out an effective crop rotation model suitable for morel and (ii) to uncover the alleviating effect and underlying mechanism of crop rotation on the continuous cropping obstacles of morel, ultimately providing theoretical support and technical references for the sustainable production of morel.

## 2. Materials and Methods

### 2.1. Experimental Design

The experiment was carried out from November 2023 to April 2025 in a greenhouse situated in the Tongxin Dryland Water-saving and High-efficiency Agricultural Science and Technology Park, Wuzhong City, Ningxia, China (105°54′24″ E, 36°58′48″ N). The experimental area within the greenhouse measured 8 m × 63 m. At the onset of the experiment, the soil used for cultivating morel (*Morchella esculenta* L. Liumei) was sandy loam, with the following fundamental properties: pH 8.66 (alkalinity primarily resulting from carbonates, which can decompose under acid conditions contributing to the alkaline environment), electrical conductivity (EC) 1.01 mS/cm, organic matter content of 14.83 g/kg, mineral nitrogen (N) content of 28.58 mg/kg, available potassium (K) content of 178.33 mg/kg, and available phosphorus (P) content of 24.58 mg/kg. Five treatments were established, namely morel monoculture (Control), tomato–morel rotation (TM), pepper–morel rotation (PM), watermelon–morel rotation (WM), and cabbage–morel rotation (CM). Each treatment had three replicates, and each replicate plot had dimensions of 8.0 m × 4.2 m. The plots were arranged in a randomized block design.

**Figure 1:**
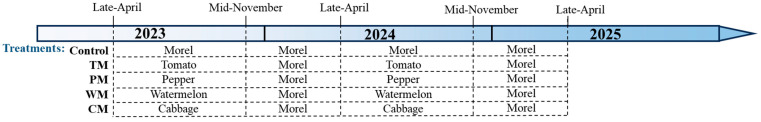
A cartoon of the timeline of the experimental process. Control, morel monoculture; TM, tomato–morel; PM, pepper–morel; WM, watermelon–morel; CM, cabbage–morel.

Morel from mushroom gill pieces was planted in mid-November each year and harvested in late April of the following year (Figure 1). Rotated crops, including tomato (*Lycopersicon esculentum* Mill. var. Yunxi), pepper (*Capsicum annum* L. var. Ningjia No. 6), watermelon (*Citrullus lanatus* var. Hualing), and cabbage (*Brassica oleracea* L. var. Zhonggan No. 21), were planted in late April of the following year and harvested in early November. Morel was directly sown in soil cultivation beds at a planting density of 120 mushrooms per square meter. The beds were 1.0 m wide and 10 cm high. After sowing, nutrient bags (polypropylene cultivation bags) were placed on the bed surface. Each nutrient bag measured 12 cm × 24 cm and contained 300 g of a mixed substrate. The dry mass ratio of the components in the substrate was wheat grains/wood chips/lime/gypsum = 76:22:1:1. This plant-based substrate was not sterilized, thus containing naturally occurring microbial communities. Nine nutrient bags were placed per square meter (each treatment had 594 replicate bags). For tomato and pepper, the planting distances were 30 cm, with row spacings of 90 cm and 50 cm, respectively. For watermelon, the planting distance was 60 cm, with row spacings of 150 cm and 60 cm, respectively. For cabbage, the planting distance was 40 cm, and the row spacing was 40 cm (equal row spacing). The planting density of morel was 120 mushrooms per square meter. For tomato, pepper, watermelon, and cabbage, the planting densities were 4.8, 4.8, 1.6, and 6.3 plants per square meter, respectively.

### 2.2. Sample Collection and Index Measurement

#### 2.2.1. Soil Sample Collection and Measurement

In 2024 and 2025, soil samples from the root zone (0–20 cm; the depth of morel stalks in the soil generally ranges from 5 to 20 cm) were collected during the peak harvest period of morel (mid-April) each year. Eight random points were selected from each plot to collect soil samples, which were then combined to form a single soil sample. Soil chemical properties were measured following the method of Sparks et al. [16]. Soil organic matter was determined using the potassium dichromate external heating method. Soil pH was measured by the potentiometric method (soil/water = 1:2.5, *w*/*w*). Soil EC was measured via the conductometer method (soil/water = 1:5, *w*/*w*). Soil mineral nitrogen (ammonium N + nitrate N) was determined using a continuous flow analyzer. Soil available P was measured by the molybdenum blue colorimetric method, and soil available K was determined by the flame photometer method.

The activities of morel-cultivated soil urease (catalytic reaction/urea hydrolysis; substrate/urea), sucrase (catalytic reaction/carbohydrate hydrolysis; substrate/sucrose), alkaline phosphatase (catalytic reaction/phenylphosphate hydrolysis; substrate/phenylphosphate), catalase (catalytic reaction/coupled oxidation; substrate/hydrogen peroxide), and polyphenol oxidase (catalytic reaction/phenol oxidation; substrate/pyrogallol) were determined following the methods described by Tian et al. [17]. Morel-cultivated soil alkaline protease (catalytic reaction/amide bond hydrolysis; substrate/casein) was measured using the method described by Jesmin et al. [18].

Phenolic compounds (comprising 16 phenolic acid compounds, 3 flavonoids, and 4 other compounds) were determined following the method described by Li et al. [19]. Briefly, samples were pre-treated and analyzed by UPLC-MS, and phenolic acids were identified based on accurate molecular weight and MS/MS fragmentation patterns. Amino sugars (glucosamine, mannosamine, muramic acid, and galactosamine), which serve as specific indicators of fungal and bacterial residues due to their presence in the cell walls of fungi and bacteria [20], were determined using the method of Zhang and Amelung [21]. Specifically, glucosamine and muramic acid are primarily found in bacterial peptidoglycan, while mannosamine and galactosamine are often associated with fungal chitin. These sugars serve as biomarkers for microbial contamination or decomposition. Additionally, toxic metals and metalloids (such as As, Cd, Al, Cr, and Pb) were measured using inductively coupled plasma atomic emission spectrometry (ICP-AES) [22].

#### 2.2.2. Mycelial Growth Rate, Individual Mushroom Weight, and Morel Yields

The bags were fully colonized by morels in early February. Primordia started to emerge in late February, and harvesting commenced in mid-March, concluding in mid-April. Within each plot, 6 points were selected to measure mycelial growth. The mycelial extension length of *Morchella* was recorded using a ruler every day, and continued recording until the mycelial growth became stable. Take the average value of the daily growth amounts from the 6 replicates in each plot as the daily mycelial growth amount of that plot, and finally convert it to the mycelial growth rate (mm/d). The morel yield was determined by measuring the entire fruiting body of the morel, including both the cap and the stalk. To avoid ambiguity, we focused on the mature specimens, where the cap was fully expanded and the stalk was of full length. For each cultivation unit (e.g., each bag mentioned in the yield determination description), record the individual weight of every mature morel harvested in each collection batch during the specified harvest period. The yield from each collection was recorded by summing up the total weight of mature morels harvested from each bag during the specified harvest period.

#### 2.2.3. Soil Quality Assessment

Soil quality was comprehensively evaluated by calculating the soil quality index area (SQI-area) [23]. Initially, key soil indicators that were significantly correlated with the morel yield (including EC, organic matter, available P, alkaline phosphatase, catalase, polyphenol oxidase, alkaline protease, phthalic acid, benzoic acid, sinapic acid, vanillin, glucosamine, and mannosamine) were transformed into dimensionless values ranging from 0 to 1. For indicators that were significantly (*p* < 0.05) positively correlated with the morel yield, the formula *y* = (*X_i_* − *X_min_*)/(*X_max_* − *X_min_*) was employed, where *y* and *X_i_* represent the standardized value and the measured value of soil quality indicator i, respectively, and *X_min_* and *X_max_* denote the minimum and maximum measured values of the key soil quality indicators. For indicators that were significantly (*p* < 0.05) negatively correlated with the morel yield, the formula *y* = 1 − (*X_i_* − *X_min_*)/(*X_max_* − *X_min_*) was used. Moreover, for EC (threshold: 0.8–1.5), the method proposed by Zheng et al. was utilized for calculation [24]. Finally, the soil quality index was calculated according to the formula SQI-area = $0.5\sum_{i}^{n}S_{i}^{2}sin\frac{2\pi }{n}$ [25].

### 2.3. Data Statistics and Analysis

The experimental data were collated using Microsoft Excel 2019 software. One-way ANOVA was then performed on the experimental data with SPSS Statistics 25 software. The Tukey method was employed for multiple comparisons, and the significance of differences was examined at the *p* < 0.05 level.

## 3. Results

### 3.1. Soil Chemical Properties

**Figure 2:**
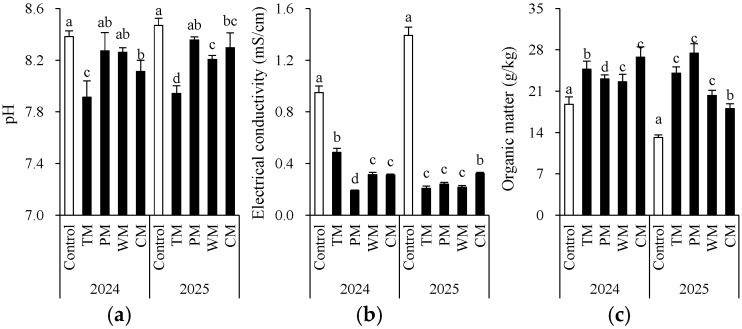
Morel root-zone soil pH (**a**), electrical conductivity (**b**), and organic matter (**c**) as affected by different treatments. The values are expressed as mean ± standard deviation. For the same year, different letters denote significant differences at the *p* < 0.05 level. Control, morel monoculture; TM, tomato–morel; PM, pepper–morel; WM, watermelon–morel; CM, cabbage–morel. Each treatment had three replicates, and each replicate contained six morel soils. In 2025, the soil from 2024 was replanted in the same way.

**Figure 3:**
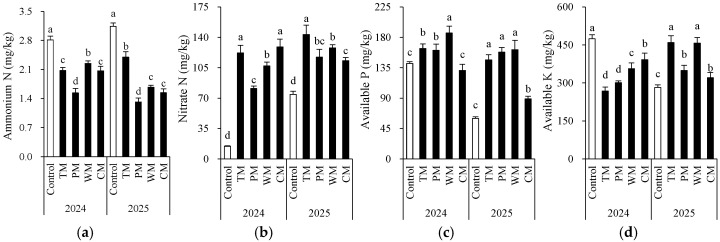
Morel root-zone soil ammonium N (**a**), nitrate N (**b**), available P (**c**), and available K (**d**) as affected by different treatments. The values are expressed as mean ± standard deviation. For the same year, different letters denote significant differences at the *p* < 0.05 level. Control, morel monoculture; TM, tomato–morel; PM, pepper–morel; WM, watermelon–morel; CM, cabbage–morel.

Each rotation treatment had a significant impact on soil fertility indicators (Figure 2 and Figure 3). When compared with the control, all rotation treatments demonstrated better performance in multiple soil chemical properties. Specifically, in 2024, the TM and CM treatments had significantly higher contents of available N (155.5 mg/kg and 147.4 mg/kg), nitrate N (122.0 mg/kg and 128.9 mg/kg), and organic matter (24.7 g/kg and 26.8 g/kg) than the control. The WM treatment had the highest available P content (186.4 mg/kg). In 2025, the TM, PM, and WM treatments still maintained high levels of available N, nitrate N, and available P. In particular, the TM treatment had 169.3 mg/kg of available N and 143.3 mg/kg of nitrate N, which were significantly higher than those of the control.

From an interannual perspective, the morel mono-culture soil (control) exhibited a trend of increasing pH, increasing EC, and decreasing content of some nutrients, suggesting that continuous cropping might lead to intensified soil alkalization and salinization. However, the rotation treatments generally presented lower EC values. For instance, in 2025, the TM, PM, and WM treatments all had values about 0.2 mS/cm, which were significantly lower than that of the control (1.4 mS/cm). This indicates that rotation effectively mitigated soil salt accumulation. Additionally, except for the PM treatment, most rotation treatments decreased the soil ammonium N content. The significant differences in the morel monoculture soil (control) between 2024 and 2025 are mainly attributed to the use of fresh substrates with different initial properties in the two years. In conclusion, introducing tomato, pepper, watermelon, or cabbage into the rotation system can significantly improve the soil chemical environment, enhance nutrient availability, and alleviate soil degradation caused by continuous cropping.

### 3.2. Soil Enzyme Activity

**Figure 4:**
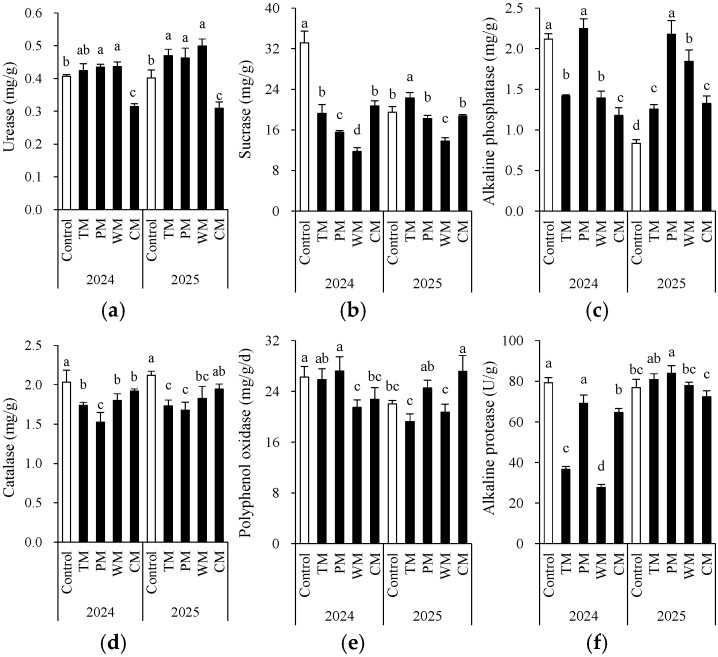
Morel root-zone soil urease (**a**), sucrase (**b**), alkaline phosphatase (**c**), catalase (**d**), polyphenol oxidase (**e**), and alkaline protease (**f**) as affected by different treatments. The values are expressed as mean ± standard deviation. For the same year, different letters denote significant differences at the *p* < 0.05 level. Control, morel monoculture; TM, tomato–morel; PM, pepper–morel; WM, watermelon–morel; CM, cabbage–morel. Enzyme activities are expressed per gram (g) of fresh weight in the text.

Each rotation treatment exerted a significant influence on soil enzyme activity (Figure 4). When compared with the control, all rotation treatments exhibited notable differences in various enzyme activity indicators. In 2024, the sucrose enzyme activity in the WM treatment (11.8 mg/g) was significantly lower than that in the control (33.2 mg/g). Conversely, the PM treatment demonstrated higher alkaline phosphatase (2.2 mg/g) and polyphenol oxidase (27.2 mg/g) activities compared to the control. In 2025, the PM treatment had significantly higher alkaline phosphatase (2.2 mg/g) and alkaline protease (83.9 U/g) activities than the other treatments. This indicates that pepper rotation may contribute to enhancing the soil’s P transformation and protein degradation capabilities.

Overall, based on the two repeats of one harvest cycle, the impact of different previous crop species on soil enzyme activity exhibited interannual variations, yet some trends remained consistent. For instance, in both years, the WM treatment significantly decreased the activity of the sucrose enzyme. In contrast, the CM treatment led to lower urease activity in 2024 and 2025. On the other hand, compared to the control, the TM and PM treatments demonstrated better or comparable enzyme activities in most indicators, indicating that tomato and pepper, as previous crop species, might contribute to maintaining or enhancing soil biochemical activity. Additionally, the catalase activity was generally higher in the control but decreased in rotation treatments, suggesting that monoculture may result in increased soil oxidative stress. However, due to the fact that the roots of different plants break or release root cap cells in different ways, there was some variability in soil enzymes between the two years (the trends among treatments were not consistent). This variability confounded the interpretation of enzyme activities.

### 3.3. Soil Phenolic Compounds

Different rotation treatments significantly influenced the content and composition of phenolic compounds in the soil (Tables S1 and S2). All four rotation treatments reduced the accumulation of most phenolic compounds in the soil compared to the control (Tables S1 and S2). In 2024, the control showed the highest contents of toxic phenolic acids (e.g., phthalic acid, benzoic acid), while the WM treatment exhibited the lowest levels of these compounds; in 2025, the CM treatment further reduced the contents of eugenol (90.4 ng/g) and ferulic acid (129.9 ng/g) compared to the control. Notably, the PM treatment showed increased contents of epicatechin and vanillin in 2025, indicating crop-specific effects of rotation on soil phenolic acid pools.

In the morel monoculture soil (control), phenolic acids remain in a constantly high accumulation state. Specifically, the contents of known morel-toxic substances such as phthalic acid and benzoic acid are significantly higher than those in the rotation system. Over the two-year period, the WM and CM treatments maintained low levels of most phenolic acid indicators. This reflects that watermelon and cabbage, as previous crop species, have a greater potential for alleviating phenolic acid-related disorders, since phenolic acid substances (e.g., phthalic acid, benzoic acid, and ferulic acid) have been shown to inhibit the growth of *Morel mycelia*. In addition, polyhydroxyphenolic acids (e.g., gallic acid, protocatechuic acid, catechol) exhibit strong metal-chelating capabilities through coordination interactions between their multiple hydroxyl groups and metal ions (such as Fe^3+^ and Cu^2+^), forming stable complexes. Meanwhile, flavonoids (e.g., catechin, epicatechin, rutin, kaempferol) demonstrate significant binding affinity toward various metal ions, including Zn^2+^ and Pb^2+^.

**Figure 5:**
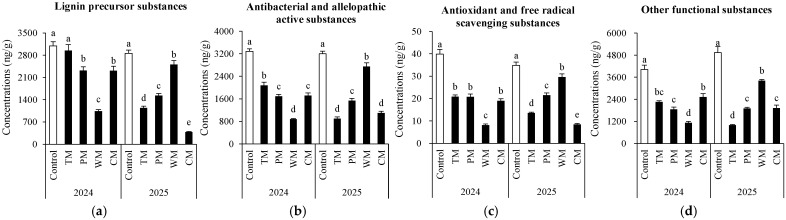
Potential functions of soil phenolic compounds under different treatments. (**a**) Lignin precursor substances (syringic acid, ferulic acid, p-Coumaric acid, protocatechuic aldehyde, eugenol, and sinapic acid), (**b**) antibacterial and allelopathic active substances (gallic acid, protocatechuic acid, p-coumaric acid, catechol, benzoic acid, p-hydroxybenzoic acid, vanillic acid and vanillin), (**c**) antioxidant and free radical scavenging substances (caffeic acid, kaempferol, epicatechin, catechin, chlorogenic acid and rutin) and (**d**) other functional substances (quinic acid, phthalic acid and oleanolic acid). The values are expressed as mean ± standard deviation. For the same year, different letters denote significant differences at the *p* < 0.05 level. Control, morel monoculture; TM, tomato–morel; PM, pepper–morel; WM, watermelon–morel; CM, cabbage–morel. Each treatment had three replicates, and each replicate contained six morel soils.

Soil phenolic compounds were further classified according to potential functions (Figure 5). Overall, compared to most other treatments, the control demonstrated significantly higher levels of the four substances in both years. Moreover, the WM treatment exhibited the most notable annual variation. In 2024, it strongly inhibited all four substances, resulting in the lowest content. However, in 2025, it promoted three of the substances. The TM treatment had a weak inhibitory effect in 2024 but shifted to a strong inhibitory effect in 2025. The PM treatment showed a relatively stable inhibitory effect over the two-year period, with medium to low content levels. The CM treatment had a moderate inhibitory effect in 2024. Nevertheless, in 2025, its inhibitory effect on lignin precursors and antioxidant compounds increased significantly.

In summary, crop rotation can reduce the accumulation of toxic phenolic acids in the soil and offer an effective approach to alleviating the continuous cropping disorder of morel.

### 3.4. Soil Amino Sugars

**Figure 6:**
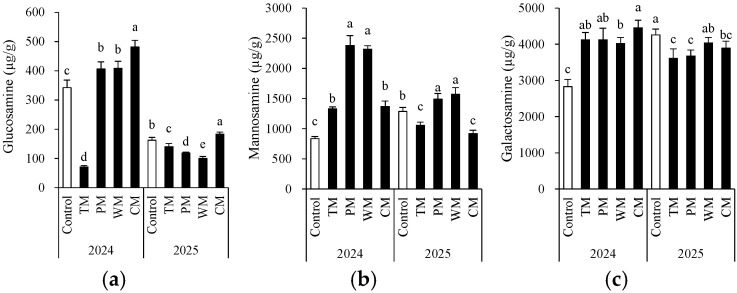
Morel root-zone soil glucosamine (**a**), mannosamine (**b**), and galactosamine (**c**) as affected by different treatments. The values are expressed as mean ± standard deviation. For the same year, different letters denote significant differences at the *p* < 0.05 level. Control, morel monoculture; TM, tomato–morel; PM, pepper–morel; WM, watermelon–morel; CM, cabbage–morel.

The rotation pattern had a significant impact on the accumulation of the three types of amino sugars, and obvious interannual differences were observed (Figure 6). Regarding glucosamine, in 2024, the CM treatment exhibited the highest content (481.2 μg/g), while the control had a lower content. In 2025, the overall content decreased, yet the CM treatment still maintained a relatively high level. The TM and PM treatments showed a significant decrease. Notably, the total glucosamine content in 2025 was lower than that in 2024. This could be associated with annual environmental variations. Specifically, the extended period of low temperatures during the growing season in 2025 led to a decreased accumulation of glucosamine. The content of galactosamine was highest in the CM treatment in 2024 and in the control in 2025. Concerning mannosamine, in both years, the PM and WM treatments had the highest content. In 2024, the PM treatment reached 2377.7 μg/g. In 2025, the WM and PM treatments were still significantly higher than other groups, suggesting that the rotation of solanaceous and gourd crops might be more conducive to the accumulation of mannosamine. The TM and PM treatments generally had lower contents over the two years. These findings indicate that the type of crop rotation significantly regulates the composition and dynamics of soil microbial residue carbon (as indicated by amino sugars).

### 3.5. Soil Toxic Metals and Metalloids

**Figure 7:**
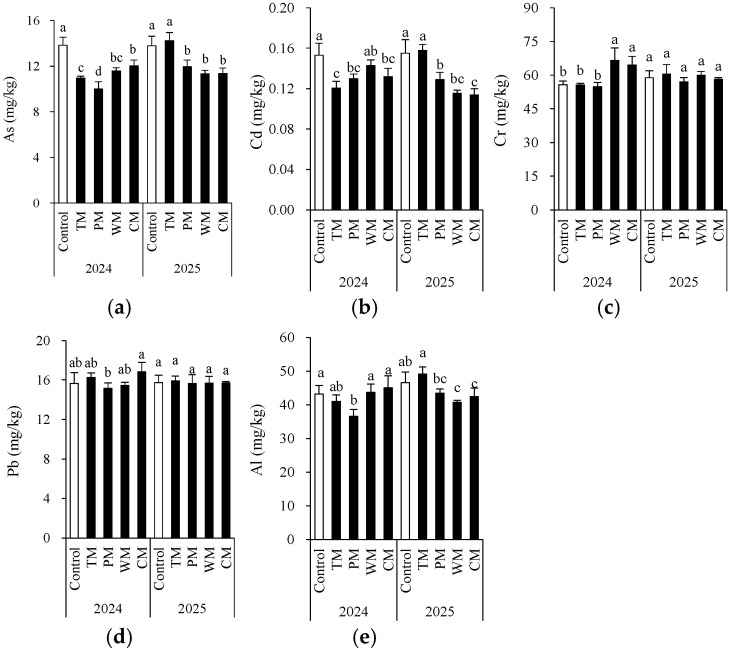
Morel root-zone soil As (**a**), Cd (**b**), Cr (**c**), Pb (**d**), and Al (**e**) as affected by different treatments. The values are expressed as mean ± standard deviation. For the same year, different letters denote significant differences at the *p* < 0.05 level. Control, morel monoculture; TM, tomato–morel; PM, pepper–morel; WM, watermelon–morel; CM, cabbage–morel.

Crop rotation significantly modified the contents of certain toxic metals and metalloids (Figure 7). Generally, compared with the control, all four rotation treatments decreased the contents of As and Cd in most instances. Particularly in the PM treatment in 2024, the As content decreased significantly to 10.0 mg/kg, demonstrating a better mitigating effect. The Al content remained at a relatively low level in the PM treatment for two consecutive years, indicating that pepper rotation may contribute to reducing Al accumulation. In different rotation systems, the changes in Cr and Pb were more intricate. In 2024, the Cr content in the WM and CM treatments was significantly higher than that of the control, while in 2025, there was no significant difference among the treatments. This indicates that the influence of the year factor on Cr may be greater than that of crop rotation itself. The Pb content remained relatively stable over the two-year period. Most of the differences among the treatments were not statistically significant, indicating that crop rotation had a limited impact on Pb accumulation. Notably, according to China’s risk screening values for soil contamination of agricultural land (GB 15618-2018) [26], for all treatment, the toxic metal levels were below the screening value (for pH > 7.5, As: 25 mg/kg; Cd: 0.6 mg/kg; Cr: 250 mg/kg; Pb: 170 mg/kg).

In general, the pepper–morel rotation (PM) was more effective in reducing As and Cd levels. However, the effects of different rotation patterns on Al and Cr varied from year to year, suggesting that the impact of crop rotation is jointly regulated by environmental factors and crop types.

### 3.6. Mycelial Growth Rate, Individual Mushroom Weight, Morel Yields, and Their Association with Soil Properties

**Figure 8:**
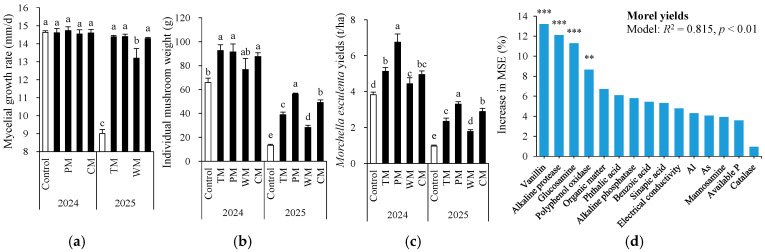
Mycelial growth rate (**a**), individual mushroom weight (**b**), and morel yields (**c**) as affected by different treatments and random forest modeling analysis revealing the importance of main predictors (% increase in MSE) for morel yields (**d**). The values are expressed as mean ± standard deviation. For the same year, different letters denote significant differences at the *p* < 0.05 level. Control, morel monoculture; TM, tomato–morel; PM, pepper–morel; WM, watermelon–morel; CM, cabbage–morel. *R*^2^ denotes the proportion of variance explained. ** and *** indicate *p* < 0.01 and *p* < 0.001, respectively.

In 2024, there was no significant difference in mycelial growth rate across all treatments (Figure 8a). However, in 2025, the control had the lowest rate. TM and PM achieved the highest rate, followed by CM and WM, proving that all rotation treatments effectively promoted mycelial growth compared to the control. With respect to individual mushroom weight (Figure 8b), compared to the control, it was significantly increased by TM, CM, and PM in 2024, and by all rotation treatments in 2025. The morel yield in the rotation treatment was notably higher than that in the monoculture control (Figure 8c). In 2024, the morel yields of the TM, PM, WM, and CM treatments were 34.3%, 76.7%, 16.2%, and 29.6% higher than the control, respectively. In 2025, the yield increases for each treatment were 142.3%, 241.2%, 83.5%, and 197.9%, respectively. Overall, the PM treatment exhibited the highest morel yield increase rate over the two-year period. Particularly in 2025, the morel yield increase was especially prominent, indicating that the pepper–morel rotation system has a significant and stable advantage in promoting the yield of morels.

The correlation analysis among all soil properties is shown in Table S3. The morel yield is significantly correlated with several soil properties (Table 1). Among these, soil organic matter (*r* = 0.570, *p* < 0.001), available P (*r* = 0.544, *p* < 0.01), and polyphenol oxidase activity (*r* = 0.533, *p* < 0.01) show extremely significant positive correlations with the morel yield. Conversely, soil As content (*r* = −0.561, *p* < 0.001), catalase activity (*r* = −0.496, *p* < 0.01), and aluminum content (*r* = −0.477, *p* < 0.01) show extremely significant negative correlations with the yield. Additionally, EC (*r* = −0.405, *p* < 0.05), alkaline phosphatase (*r* = 0.425, *p* < 0.05), vanillin (*r* = 0.714, *p* < 0.001), and glucosamine (*r* = 0.600, *p* < 0.001) also exhibit significant correlations. This indicates that soil nutrients, enzyme activities, heavy metal contents, and certain phenolic acids and amino sugar substances are important soil properties influencing the yield of morels.

To further confirm key soil indicators for predicting morel yields, random forest (RF) modeling analysis was conducted for soil properties that were significantly correlated with morel yields (Figure 8d). As a result, the RF models explained 81.5% of the variance in the morel yields with vanillin, alkaline protease, glucosamine, and polyphenol oxidase being the most important predictors (all *p* < 0.01).

### 3.7. Overall Soil Quality Index and Its Association with Morel Yields

**Figure 9:**
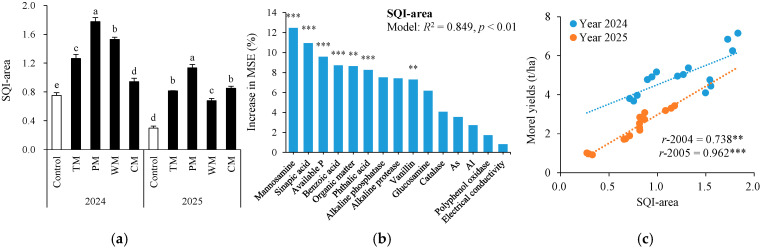
Soil quality index area (SQI-area) under different treatments (**a**), random forest modeling analysis revealing the importance of main predictors (% increase in MSE) for SQI-area (**b**), and relationships between SQI-area and morel yields (**c**). The values are expressed as mean ± standard deviation. For the same year, different letters denote significant differences at the *p* < 0.05 level. Control, morel monoculture; TM, tomato–morel; PM, pepper–morel; WM, watermelon–morel; CM, cabbage–morel. *R*^2^ denotes the proportion of variance explained. ** and *** indicate *p* < 0.01 and *p* < 0.001, respectively.

Based on the key soil factors closely associated with the morel yield (Table 1), the soil comprehensive quality index (Figure 9a) was computed. Different rotation treatments exhibited significant improvement effects on the soil comprehensive quality index (SQI-area). In 2024, the SQI-area values of the TM, PM, WM, and CM treatments were 68.0%, 137.3%, 104.0%, and 25.3% higher than those of the control, respectively. In 2025, the increase in SQI-area was even more notable for each rotation treatment. Specifically, the TM, PM, WM, and CM treatments were 173.3%, 276.7%, 126.7%, and 183.3% higher than the control, respectively. To further confirm key soil indicators for predicting SQI-area, random forest (RF) modeling analysis was conducted for soil properties (Figure 9b). As a result, the RF models explained 84.9% of the variance in the morel yields with manosamine, sinapic acid, available P, benzoic acid, organic matter, phthalic acid, and vanillin being the most important predictors (all *p* < 0.01).

Furthermore, in 2024 and 2025, the SQI-area was significantly and positively correlated with the morel yield (*r*-2024 = 0.738, *p* < 0.01; *r*-2025 = 0.962, *p* < 0.001; Figure 9c). These findings suggest that the rotation pattern effectively enhanced the soil comprehensive quality in different years, thereby boosting the morel yield. Among these, the pepper–morel rotation demonstrated the greatest improvement over the two-year period, indicating its prominent potential for maintaining and enhancing soil comprehensive quality.

## 4. Discussion

This study systematically analyzed the effects of different rotation patterns on the soil micro-ecosystem and the morel yield. It revealed the multi-pathway mechanism by which rotation alleviates the problems associated with continuous cropping. Compared with monoculture, the rotation treatments significantly enhanced the soil chemical properties, enzyme activities, and microbial residue composition. They also effectively reduced the accumulation of plant-toxic substances, thus improving the soil comprehensive quality index (SQI-area) and the yield of morels.

Firstly, the rotation had a particularly notable impact on improving the soil chemical environment. Continuous cropping led to an increase in soil pH and EC, indicating the risks of alkalization and salinization. However, the rotation treatments (especially TM and PM) significantly decreased the EC and increased the contents of organic matter and available P. The EC effect is correlated with root masses of the alternative crop. This result is consistent with the studies of Wang et al. [27] and Jalli et al. [28], suggesting that rotation promotes soil nutrient cycling and availability by introducing different crop root exudates and residues. For instance, tomatoes and peppers may activate phosphorus through the release of specific organic acids, while watermelons help maintain a low-salt environment [29,30,31].

Secondly, soil enzyme activities through rotation further optimized the soil biochemical functions. In this research, the PM treatment demonstrated remarkable performance in alkaline phosphatase and protease activities. Alkaline phosphatase is a key hydrolase that catalyzes the hydrolysis of organophosphorus compounds in soil into inorganic phosphorus (e.g., PO_4_^3−^), which is directly absorbable by plants—thus, the higher alkaline phosphatase activity under PM directly reflects an enhanced capacity of soil to transform organophosphorus into available phosphorus. Meanwhile, protease mainly decomposes soil organic nitrogen (e.g., proteins, peptides) into amino acids, which are critical intermediates for soil nitrogen metabolism; the elevated protease activity under PM therefore indicates strengthened decomposition of organic nitrogen and improved nitrogen supply potential in soil [32]. Conversely, the peroxidase activity in continuously cropped soil remained consistently high. This might reflect the accumulation of oxidative stress in the soil, as peroxidase is a key enzyme that scavenges reactive oxygen species (ROS) produced under stress (e.g., allelochemical accumulation, nutrient imbalance in continuous cropping). In contrast, peroxidase activity in rotation treatments decreased to levels closer to that of healthy reference soil, a trend suggesting soil health restoration [33]. This finding aligns with the research of Zhang et al. [34] regarding the correlation between soil enzyme activity and microbial community function. Rotation may improve the soil micro-ecosystem by altering the microbial community structure and regulating key enzyme activities, thus restoring the soil micro-ecological balance.

Regarding allelochemicals, the accumulation of phenolic acid substances (such as phthalic acid, benzoic acid, and ferulic acid) in continuous cropping soil (control) was significantly greater than that in rotation treatments. These substances have been demonstrated to inhibit the growth of morel [35]. The WM and CM treatments exhibited stable performance in reducing phenolic acid content. This might be associated with the stimulation of phenolic acid-degrading microorganisms by the root exudates of fruiting crops and cruciferous vegetables [36,37]. The reduced accumulation of toxic phenolic acids in rotation treatments may be associated with altered soil microbial activity (e.g., proliferation of phenolic acid-degrading microbes stimulated by rotational crop root exudates) [38], but the specific process (e.g., enhanced degradation or reduced input) cannot be confirmed by the current snapshot data.

Soil organic acids (OAs) involved in the Krebs cycle (e.g., citric acid, malic acid, succinic acid)—key mediators of metal chelation and enzyme activity regulation—are closely linked to this metabolic network. Notably, these Krebs cycle OAs serve as preferred carbon substrates for numerous soil microbes, meaning their degradation or accumulation can exert huge effects on soil microbial community structure and metabolic activity—two factors that directly influence the turnover of phenolic substances like vanillin. Although we did not directly assay Krebs cycle OAs in this study, the specific regulation of vanillin and other phenolic substances by PM treatment implies potential shifts in the soil OA pool. This link may involve two key processes: first, pepper-microbe interactions may alter the secretion or microbial degradation of Krebs cycle OAs; second, such changes in OA levels can adjust the bioavailability of metal ions—some of which act as cofactors for enzymes involved in phenolic acid synthesis, thereby further shaping the metabolic flux of phenolic acids. It is important to clarify the connection between the Krebs cycle (tricarboxylic acid cycle) and vanillin biosynthesis here. Vanillin is primarily synthesized via the shikimic acid pathway in plants and microbes, which relies on carbon skeletons and energy. The Krebs cycle contributes to this process by regulating carbon partitioning in soil ecosystems: shifts in Krebs cycle OA pools (e.g., increased/decreased citrate or malate) can redirect the flow of carbon intermediates—either supplying more carbon to the shikimic acid pathway for phenolic synthesis or diverting carbon toward microbial OA decomposition, indirectly affecting vanillin production. Future studies focusing on Krebs cycle OA quantification, their microbial degradation dynamics, and their dual associations with soil metals—specifically, OA chelate metal ions that act as cofactors for enzymes, especially reactive oxygen species (ROS) decomposers—and carbon partitioning to the shikimic acid pathway will help clarify this indirect regulatory pathway.

Rotation also had a significant impact on the composition and dynamics of soil amino sugars. The content of mannosamine was higher in the PM and WM treatments, suggesting that more fungal-derived residues were input. This could be related to the promotion of fungal community proliferation by the root systems of solanaceous and fruiting crops [20]. Notably, while our study focused on the overall increase in fungal residues (indicated by elevated mannosamine) under PM and WM treatments, we did not specifically investigate the potential antagonistic interactions between soil fungi (e.g., the proliferating fungi promoted by pepper/watermelon roots) and the morel (the target fungal crop in this system). Antagonism between soil fungi and morels—such as competition for nutrients (e.g., carbon, nitrogen) or the secretion of inhibitory metabolites—would be a critical factor in understanding the actual ecological role of increased fungal residues. If antagonistic interactions exist, the elevated fungal community might limit morel growth; if not, the increased fungal residues could instead contribute to soil organic matter accumulation without adverse effects on morels.

The highly significant positive correlation between the soil quality index (SQI-area) and the yield of morels (Figure 2) suggests that SQI-area can serve as a comprehensive indicator for evaluating the effectiveness of rotation patterns in alleviating issues stemming from continuous cropping [23,25]. The pepper–morel rotation (PM) demonstrated the best performance in enhancing SQI-area and the morel yield, indicating that this rotation mode has notable advantages and application potential in regulating the soil micro-ecosystem and alleviating continuous cropping problems. This research provides an important theoretical foundation for the green control of morel growth obstacles caused by continuous cropping. Future studies can further integrate microbial community structure analysis and metabolomics techniques to deeply uncover the molecular mechanism by which rotation modulates the soil micro-ecosystem.

A key feature of the rotation system is the absence of morels in one growing season (e.g., pepper/tomato/watermelon/cabbage planting from April to November). This absence may reduce the input of morel-specific exudates (e.g., phenolic acids) into the soil, while soil microbes (e.g., phenolic acid-degrading bacteria/fungi) continue to decompose residual phenolic compounds—this two-fold effect (reduced input + continuous microbial decomposition) may contribute to the lower phenolic acid contents in rotation treatments compared to the monoculture control. However, the current experimental design cannot quantify the relative contributions of ‘reduced input’ and ‘microbial decomposition’; future studies could compare phenolic acid dynamics in ‘rotation without morels’ vs. ‘fallow without morels’ to isolate these effects.

## 5. Conclusions

The rotation cultivation method can remarkably enhance the overall soil quality and effectively mitigate the morel growth obstacles caused by continuous cropping. The rotation effect is attained through multiple channels: (a) improving the chemical properties of the soil, alleviating salinization, and increasing the contents of organic matter and available P; (b) strengthening the nutrient cycling capacity and reducing the accumulation of morel-toxic phenolic acids; (c) altering the composition of microbial residues (amino sugars), indicating an improvement in the health status of the soil micro-ecosystem. Compared with the morel monoculture, the four rotation patterns have significantly improved the soil quality index (SQI-area) over a two-year period. Among them, the pepper–morel rotation (PM) pattern exhibits the most prominent and stable improvement effect and is the optimal mode for achieving high morel yields and the coordinated development of soil health. This study only measured soil properties at the morel harvest stage (snapshot data), so the specific processes of phenolic acid dynamics (synthesis vs. degradation) and the relative contributions of ‘reduced morel exudates’ vs. ‘microbial decomposition’ remain to be explored in future studies with dynamic sampling designs.

## Figures and Tables

**Table 1:** Pearson correlation coefficient (*r*) between morel yields and root-zone soil properties.

Soil Properties	Coefficient (*r*)	Soil Properties	Coefficient (*r*)	Soil Properties	Coefficient (*r*)
pH	−0.216	Al	−0.477 **	Protocatechuic aldehyde	0.007
Electrical conductivity	−0.405 *	Cr	−0.049	Syringaldehyde	0.103
Organic matter	0.570 ***	Pb	0.067	Sinapic acid	−0.364 *
Ammonium N	−0.336	Syringic acid	−0.151	Kaempferol	0.038
Nitrate N	−0.076	Ferulic acid	0.116	Epicatechin	0.203
Available P	0.544 **	Quinic acid	0.061	Catechin	0.080
Available K	−0.249	Caffeic acid	−0.274	Chlorogenic acid	−0.152
Urease	−0.149	Gallic acid	0.269	Rutin	−0.236
Sucrose	−0.062	Phthalic acid	−0.431 *	Vanillic acid	−0.101
Alkaline phosphatase	0.425 *	Protocatechuic acid	−0.317	Oleanolic acid	−0.068
Catalase	−0.496 **	p-Coumaric acid	0.308	Vanillin	0.714 ***
Polyphenol oxidase	0.533 **	o-Coumaric acid	−0.060	Glucosamine	0.600 ***
Alkaline protease	−0.462 *	Catechol	0.030	Manosamine	0.526 **
As	−0.561 ***	Benzoic acid	−0.446 *	Galactosamine	0.152
Cd	−0.188	p-Hydroxybenzoic acid	0.004		

* *p* < 0.05, ** *p* < 0.01, *** *p* < 0.001. For the Pearson correlation analysis, a total of 30 soil samples were used, and the analysis integrated data collected from both the 2024 and 2025 growing seasons. The correlation analysis among all soil properties is shown in Table S3.

## Data Availability

The data presented in this study are available in the article.

## Supplementary Materials

The following supporting information can be downloaded at: https://www.mdpi.com/article/10.3390/plants14213317/s1, Table S1. Soil phenolic compounds as affected by different treatments in 2024. Table S2. Soil phenolic compounds as affected by different treatments in 2025. Table S3. Pearson correlation coefficient (r) between soil properties.

## References

[1] Sunil C., Xu B. (2022). Mycochemical profile and health-promoting effects of morel mushroom *Morchella esculenta* (L.)—A review. Food Res. Int..

[2] Wu H., Chen J., Li J., Liu Y., Park H.J., Yang L. (2021). Recent advances on bioactive ingredients of *Morchella esculenta*. Appl. Biochem. Biotechnol..

[3] Shi X., Liu D., He X., Liu W., Yu F. (2022). Epidemic identification of fungal diseases in *Morchella* cultivation across China. J. Fungi.

[4] Liu W., He P., Shi X., Zhang Y., Perez-Moreno J., Yu F. (2023). Large-scale field cultivation of *Morchella* and relevance of basic knowledge for its steady production. J. Fungi.

[5] Dong H., Yu H., Zhou F., Chen H., Chen M., Zhang M., Tang J., Tang Q. (2024). Development status and trend of rare edible mushrooms industry in China. Edib. Med. Mushrooms.

[6] Xu L., Zhang Y., Li H., Li J., Xu J. (2024). Challenges and strategies for continuous cropping of *Morchella* spp.: A Review. Horticulturae.

[7] Liu W.Y., Guo H.B., Bi K.X., Lidiya A.S., Qi X.J., Yu X.D. (2022). Determining why continuous cropping reduces the production of the morel *Morchella sextelata*. Front. Microbiol..

[8] Li W., Li X., Zhang K., Liu J., Wei M., Yang Z., Peng Y., Zhang B. (2025). Continuous cropping obstacles in fungal production: A review of mechanisms and remedial strategies. Soil Use Manag..

[9] Osivand A., Araya H., Appiah K.S., Mardani H., Ishizaki T., Fujii Y. (2018). Allelopathy of wild mushrooms—An important factor for assessing forest ecosystems in Japan. Forests.

[10] Yin Q., Chen Z., He P.X., Liu W., Zhang W.Y., Cao X.M. (2024). Allelopathic effects of phenolic acid extracts on *Morchella* mushrooms, pathogenic fungus, and soil-dominant fungus uncover the mechanism of morel continuous cropping obstacle. Arch. Microbiol..

[11] Zhang Y., Sun S.F., Luo D.D., Mao P., Rosazlina R., Martin F.M., Xu L.L. (2023). Decline in morel production upon continuous cropping is related to changes in soil mycobiome. J. Fungi.

[12] Wang Y.W., Li S.J., Wang L.Z., Zhao Q., Bai M.Q., Naicker O., Li Q., Zhang C.L. (2024). Cobweb disease on *Morchella sextelata* caused by *Hypomyces* spp. in Sichuan province, China. Crop Prot..

[13] Dury J., Schaller N., Garcia F., Reynaud A., Bergez J.E. (2012). Models to support cropping plan and crop rotation decisions. A review. Agron. Sustain. Dev..

[14] Shah K.K., Modi B., Pandey H.P., Subedi A., Aryal G., Pandey M., Shrestha J. (2021). Diversified crop rotation: An approach for sustainable agriculture production. Adv. Agric..

[15] Su D.W., Song F.F., Luo H.L., Lin H., Lin D.M., Liu P.H., Lin X.S., Lin Z.X., Zhang L.L., Lu G.D. (2022). Effect of different rotation systems on production and quality of black morel (*Morchella importuna*). Agronomy.

[16] Sparks D.L., Page A.L., Helmke P.A., Loeppert R.H., Soltanpour P.N., Tabatabai M.A., Johnston C.T., Sumner M.E. (1996). Methods of Soil Analysis, Part 3–Chemical Methods.

[17] Tian Y., Zhang X., Wang J., Gao L. (2013). Soil microbial communities associated with the rhizosphere of cucumber under different summer cover crops and residue management: A 4-year field experiment. Sci. Hortic..

[18] Jesmin T., Margenot A.J., Mulvaney R.L. (2022). A comprehensive method for casein-based assay of soil protease activity. Commun. Soil Sci. Plant Anal..

[19] Li J., Huang Y., Chen L., Gao S., Zhang J., Zhang D. (2023). Understory plant diversity and phenolic allelochemicals across a range of Eucalyptus grandis plantation ages. J. For. Res..

[20] Joergensen R.G. (2018). Amino sugars as specific indices for fungal and bacterial residues in soil. Biol. Fertil. of Soils.

[21] Zhang X., Amelung W. (1996). Gas chromatographic determination of muramic acid, glucosamine, mannosamine, and galactosamine in soils. Soil Biol. Biochem..

[22] Tian Y., Chen L., Gao L., Michel F.C., Wan C., Li Y., Dick W.A. (2012). Composting of waste paint sludge containing melamine resin as affected by nutrients and gypsum addition and microbial inoculation. Environ. Pollut..

[23] Song M., Chen Q., Li X., Gao L., Tian Y. (2026). Carbon-rich amendments increase soil ecosystem multifunctionality and cucumber yields under different soil conditions. Soil Till. Res..

[24] Zheng X., Wei L., Lv W., Zhang H., Zhang Y., Zhang H., Zhang H., Zhu Z., Ge T., Zhang W. (2024). Long-term bioorganic and organic fertilization improved soil quality and multifunctionality under continuous cropping in watermelon. Agric. Ecosyst. Environ..

[25] Kuzyakov Y., Gunina A., Zamanian K., Tian J., Luo Y., Xu X., Yudina A., Aponte H., Alharbi H., Ovsepyan L. (2020). New approaches for evaluation of soil health, sensitivity and resistance to degradation. Front. Agric. Sci. Eng..

[26] (2018). Soil Environment Quality-Risk Control Standard for Soil Contamination of Agriculture Land.

[27] Wang X.Y., Duan Y., Zhang J., Ciampitti I.A., Cui J.W., Qiu S.J., Xu X.P., Zhao S.C., He P. (2022). Response of potato yield, soil chemical and microbial properties to different rotation sequences of green manure-potato cropping in North China. Soil Till. Res..

[28] Jalli M., Huusela E., Jalli H., Kauppi K., Niemi M., Himanen S., Jauhiainen L. (2021). Effects of crop rotation on spring wheat yield and pest occurrence in different tillage systems: A multi-year experiment in Finnish growing conditions. Front. Sustain. Food Syst..

[29] Li Z., Lian D., Zhang S., Yao Y., Lin B., Hong J., Wu S., Li H. (2025). Continuous cropping duration alters green pepper root exudate composition and triggers rhizosphere feedback inhibition. Agronomy.

[30] Dixon M., Simonne E., Obreza T., Liu G. (2020). Crop response to low phosphorus bioavailability with a focus on tomato. Agronomy.

[31] Kim Y.S., Kim K.H., Jeong T.G., Han J.W., Kim I.J., Kim T.I., Kim Y.H., Song Y.S. (2021). Effect of cultivation of rotation crops on soil physico-chemical properties and yield of watermelon in greenhouse. Korean J. Soil Sci. Fertil..

[32] Borase D.N., Nath C.P., Hazra K.K., Senthilkumar M., Singh S.S., Praharaj C.S., Singh U., Kumar N. (2020). Long-term impact of diversified crop rotations and nutrient management practices on soil microbial functions and soil enzymes activity. Ecol. Indic..

[33] Zhang P., Xia L., Sun Y., Gao S. (2024). Soil nutrients and enzyme activities based on millet continuous cropping obstacles. Sci. Rep..

[34] Zhang B., Li Y., Ren T., Tian Z., Wang G., He X., Tian C. (2014). Short-term effect of tillage and crop rotation on microbial community structure and enzyme activities of a clay loam soil. Biol. Fertil. Soils.

[35] Yin Q., Zhang W., Shi H., He P., Zhang F., Zhang J., Li B., Shi X., Yu F. (2024). Identification of allelochemicals under continuous cropping of Morchella mushrooms. Sci. Rep..

[36] Xu L., Wang X. (2025). A Comprehensive Review of Phenolic Compounds in Horticultural Plants. Int. J. Mol. Sci..

[37] Jin X., Wu F., Zhou X. (2020). Different toxic effects of ferulic and p-hydroxybenzoic acids on cucumber seedling growth were related to their different influences on rhizosphere microbial composition. Biol. Fertil. Soils.

[38] Jia H.T., Chen S.C., Yang S.Y., Shen Y.H., Qiao P.L., Wu F.Z., Zhou X.G. (2018). Effects of vanillin on cucumber rhizosphere bacterial community. Allelopath. J..

