# Supplementary material for: Vegetable–Mushroom Rotation Increases Morel (Morchella esculenta L.) Yields by Improving Soil Micro-Environments and Enhancing Overall Soil Quality
Source: Plants (Basel). 2025 Oct 30;14(21):3317. doi: 10.3390/plants14213317 (PMC12608601; doi:10.3390/plants14213317)
Supplement: Supplementary file 1 [file plants-14-03317-s001.zip › plants-3880585-supplementary.pdf]

Supplementary information

Table S1. Soil phenolic compounds as affected by different treatments in 2024.

Phenolic compounds	Control (morel monoculture)	TM (tomato-morel)	PM (pepper-morel)	WM (watermelon-morel)	CM (cabbage-morel)
Syringic acid (ng/g)	979.03±26.56a[1]	555.16±36.01b	443.43±35.90c	182.63±11.52e	374.73±25.51d
Ferulic acid (ng/g)	862.70±40.14a	884.86±69.73a	661.86±37.66b	374.43±26.84c	689.63±35.81b
Quinic acid (ng/g)	13.10±0.38a	0.93±0.01c	1.34±0.06b	0.64±0.03cd	0.58±0.01d
Caffeic acid (ng/g)	28.50±1.58a	12.20±0.31b	11.60±0.84b	3.34±0.29d	9.61±0.60c
Gallic acid (ng/g)	3.59±0.22a	0.79±0.04c	0.76±0.04c	1.76±0.08b	0.59±0.03c
Phthalic acid (ng/g)	4001.33±238.10a	2258±70.17b	1856±120.10c	1127.33±67.12d	2527.02±181.80b
Protocatechuic acid (ng/g)	8.57±0.29a	3.20±0.14b	3.32±0.15b	1.68±0.05d	2.36±0.18c
p-Coumaric acid (ng/g)	763.16±43.39c	1048.80±60.24a	842.53±20.98bc	311.56±12.56d	916.86±69.00b
o-Coumaric acid (ng/g)	0.25±0.00c	0.18±0.00d	0.37±0.02b	0.45±0.03a	0.40±0.01b
Catechol (ng/g)	2.44±0.10a	0.65±0.01d	1.38±0.07b	0.86±0.05c	0.73±0.03d
Benzoic acid (ng/g)	1840.60±23.35a	983.01±27.29c	833.56±39.33d	439.56±10.71e	1066.60±56.88b
p-Hydroxybenzoic acid (ng/g)	5.07±0.17a	0.77±0.03bc	0.73±0.05c	0.90±0.01b	0.70±0.03c
Protocatechuic aldehyde (ng/g)	5.28±0.12a	1.24±0.03c	1.76±0.08b	0.52±0.01d	1.64±0.12b
Syringaldehyde (ng/g)	464.60±22.01a	442.60±22.13a	362.36±22.09b	163.60±3.65d	315.06±10.85c
Sinapic acid (ng/g)	19.39±1.22a	12.26±0.92b	8.34±0.22c	4.804±0.27d	12.09±0.28b
Kaempferol (ng/g)	6.89±0.39a	3.53±0.16b	3.45±0.24b	1.24±0.06d	2.32±0.06c
Epicatechin (ng/g)	0.69±0.02b	0.28±0.01d	2.14±0.09a	0.47±0.02c	0.26±0.00d
Catechin (ng/g)	0.90±0.02d	1.00±0.05d	2.13±0.01b	1.75±0.09c	3.06±0.17a
Chlorogenic acid (ng/g)	1.54±0.11b	2.35±0.13a	0.61±0.04c	0.47±0.03c	0.55±0.03c
Rutin (ng/g)	1.24±0.06c	1.47±0.05b	0.77±0.03d	0.81±0.05d	3.14±0.17a
Vanillic acid (ng/g)	1344.60±70.60a	852.86±70.90b	628.10±27.50c	305.66±7.48e	457.60±24.40d
Oleanolic acid (ng/g)	7.43±0.41a	1.00±0.06b	0.77±0.04bc	0.46±0.02c	0.49±0.01c
Vanillin (ng/g)	78.89±1.41d	238.7±14.60a	221.56±11.50a	126.63±6.75c	188.76±9.80b

[1] The values are expressed as mean ± standard deviation. Different letters within the same row denote significant differences at the $p < 0.05$ level.

Table S2. Soil phenolic compounds as affected by different treatments in 2025.

Phenolic compounds	Control (morel monoculture)	TM (tomato-morel)	PM (pepper-morel)	WM (watermelon-morel)	CM (cabbage-morel)
Syringic acid (ng/g)	801.73±39.56a[1]	250.76±14.69d	409.06±8.70c	557.66±34.35b	90.41±7.98e
Ferulic acid (ng/g)	924.50±4.90a	336.80±19.70d	413.66±16.98c	619.26±43.81b	129.93±7.61e
Quinic acid (ng/g)	0.56±0.01c	0.87±0.02b	2.99±0.17a	0.84±0.03b	0.54±0.03c
Caffeic acid (ng/g)	28.30±1.30a	5.20±0.08d	9.97±0.30c	15.0±0.65b	2.66±0.17e
Gallic acid (ng/g)	0.20±0.01d	0.13±0.01e	0.68±0.02a	0.45±0.04b	0.34±0.01c
Phthalic acid (ng/g)	4938.33±323.70a	998.01±35.53d	1833.01±53.02c	3413.33±57.20b	1930.66±155.50c
Protocatechuic acid (ng/g)	6.72±0.49a	3.48±0.22d	5.59±0.38b	4.36±0.25c	0.94±0.04e
p-Coumaric acid (ng/g)	679.10±37.38b	357.30±17.46d	435.03±24.30c	944.56±41.43a	59.49±1.46e
o-Coumaric acid (ng/g)	0.23±0.01d	0.60±0.03b	0.81±0.07a	0.40±0.03c	0.24±0.01d
Catechol (ng/g)	1.64±0.09a	0.53±0.03c	0.76±0.03b	0.74±0.01b	0.58±0.02c
Benzoic acid (ng/g)	2141.00±66.56a	417.13±25.15d	794.16±42.12c	1783±95.66b	810.76±54.37c
p-Hydroxybenzoic acid (ng/g)	0.79±0.06b	0.58±0.03c	0.79±0.02b	1.24±0.06a	0.71±0.03b
Protocatechuic aldehyde (ng/g)	1.98±0.09a	0.64±0.03c	1.73±0.04b	2.03±0.15a	0.44±0.01d
Syringaldehyde (ng/g)	426.13±27.08a	186.80±3.46d	262.86±13.65c	369.63±2.970b	97.06±7.82e
Sinapic acid (ng/g)	22.74±1.34a	9.15±0.51c	9.56±0.41c	13.78±0.52b	2.68±0.10d
Kaempferol (ng/g)	3.54±0.03b	1.26±0.06e	2.22±0.11c	4.01±0.33a	1.58±0.09d
Epicatechin (ng/g)	0.40±0.02c	1.09±0.04b	0.36±0.01c	1.11±0.04b	1.66±0.14a
Catechin (ng/g)	1.04±0.05e	1.74±0.10c	3.46±0.29a	2.92±0.10b	1.38±0.05d
Chlorogenic acid (ng/g)	0.63±0.04c	0.49±0.02c	4.25±0.25a	3.35±0.20b	0.39±0.01c
Rutin (ng/g)	0.90±0.04d	3.73±0.06a	1.19±0.06c	3.17±0.08b	0.70±0.04e
Vanillic acid (ng/g)	976.93±10.60a	373.36±28.70d	578.76±28.60c	796.96±28.6b	185.93±4.00e
Oleanolic acid (ng/g)	1.01±0.07bc	0.64±0.03c	74.90±1.10a	1.67±0.07b	0.48±0.03c
Vanillin (ng/g)	79.29±3.62c	105.93±3.10b	154.71±8.22a	159.26±9.00a	97.73±4.11b

[1] The values are expressed as mean ± standard deviation. Different letters within the same row denote significant differences at the $p < 0.05$ level.

Table S3. Pearson correlation coefficient (r) between soil properties.

	pH	Electrical conductivity	Organic matter	Ammonium N	Nitrate N	Available P
Morel yield	-0.216	-.405*	.570**	-0.336	-0.076	.544**
pH	1	.483**	-.472**	0.16	-.564**	-0.349
Electrical conductivity	.483**	1	-.711**	.820**	-.649**	-.632**
Organic matter	-.472**	-.711**	1	-.510**	.528**	.705**
Ammonium N	0.16	.820**	-.510**	1	-.459*	-.369*
Nitrate N	-.564**	-.649**	.528**	-.459*	1	0.223
Available P	-0.349	-.632**	.705**	-.369*	0.223	1
Available K	-0.048	-0.163	0.137	0.157	-0.035	0.294

Urease	-0.062	-0.157	0.183	-0.035	0.125	.567**
Sucrase	0.097	.469**	-0.119	.505**	-.567**	-0.237
Alkaline phosphatase	0.226	-0.341	0.349	-.480**	-0.311	.568**
Catalase	.417*	.706**	-.547**	.656**	-0.322	-.575**
Polyphenol oxidase	0.331	0.053	0.068	-0.236	-.367*	-0.013
Alkaline protease	0.357	0.14	-0.183	-0.022	-0.151	-.408*
As	0.165	.549**	-0.258	.750**	-0.213	-0.34
Cd	0.205	.513**	-0.146	.782**	-0.329	-0.106
Al	0.028	0.28	0.015	.548**	0.225	-0.238
Cr	0.003	-0.143	0.224	0.176	.399*	0.193
Pb	-0.165	0.041	0.36	0.185	0.321	0.023
Syringic acid	0.35	.742**	-.384*	.581**	-.711**	-0.178
Ferulic acid	0.095	.638**	-0.191	.545**	-.474**	-0.086
Chlorogenic acid	0.319	.375*	-0.143	0.336	-.799**	0.076
Caffeic acid	.467**	.843**	-.540**	.643**	-.736**	-.366*
Gallic acid	0.278	0.283	-0.077	0.339	-.761**	0.298
Phthalic acid	.513**	.830**	-.653**	.565**	-.569**	-.551**
Protocatechuic acid	.455*	.672**	-0.316	.514**	-.677**	-0.169
p-Coumaric acid	-0.196	0.16	0.155	0.135	-0.127	0.222
o-Coumaric acid	-0.009	-.504**	.613**	-.397*	.421*	.404*
Catechol	.582**	.708**	-.483**	.581**	-.954**	-0.204
Benzoic acid	.481**	.751**	-.636**	.495**	-.542**	-.472**
p-Hydroxybenzoic acid	0.319	.422*	-0.258	.413*	-.807**	0.054
Protocatechuic aldehyde	.399*	.509**	-0.235	.393*	-.813**	-0.026
Vanillin	0.114	.553**	-0.163	.408*	-.504**	-0.017
Sinapic acid	0.295	.815**	-.433*	.705**	-.517**	-.365*
Kaempferol	0.308	.528**	-0.324	.369*	-.769**	-0.026
Epicatechin	0.091	-.362*	-0.165	-.407*	-0.049	-0.017
Catechin	0.001	-.599**	.617**	-.625**	.504**	0.353
Chlorogenic acid	0.043	-0.174	0.326	-.415*	0.134	.362*
Rutin	-.559**	-0.342	0.353	0.027	.544**	0.186
Vanillic acid	0.285	.679**	-0.341	.524**	-.707**	-0.082
Oleanolic acid	0.27	-0.156	.412*	-.415*	0.061	0.169
Vanillin	-.447*	-.502**	.649**	-.516**	.386*	.533**
Glucosamine	0.195	-0.069	0.168	0.103	-0.309	0.173
Mannosamine	0.096	-0.357	0.281	-0.303	0.126	.548**
Galactosamine	-0.081	-0.13	0.162	-0.141	.567**	-0.079
	**Available K**	**Urease**	**Sucrase**	**Alkaline phosphatase**	**Catalase**	**Polyphenol oxidase**
Morel yield	-0.249	-0.149	-0.062	.425*	-.496**	.533**
pH	-0.048	-0.062	0.097	0.226	.417*	0.331
Electrical conductivity	-0.163	-0.157	.469**	-0.341	.706**	0.053
Organic matter	0.137	0.183	-0.119	0.349	-.547**	0.068
Ammonium N	0.157	-0.035	.505**	-.480**	.656**	-0.236
Nitrate N	-0.035	0.125	-.567**	-0.311	-0.322	-.367*
Available P	0.294	.567**	-0.237	.568**	-.575**	-0.013
Available K	1	0.311	.425*	0.262	0.194	-0.309
Urease	0.311	1	-0.203	.436*	-0.342	-0.281
Sucrase	.425*	-0.203	1	0.12	.456*	0.275
Alkaline phosphatase	0.262	.436*	0.12	1	-.420*	.470**
Catalase	0.194	-0.342	.456*	-.420*	1	0.053
Polyphenol oxidase	-0.309	-0.281	0.275	.470**	0.053	1
Alkaline protease	.415*	0.102	.419*	0.261	0.191	0.021
As	.500**	0.097	.642**	-0.296	.620**	-0.292
Cd	0.328	0.209	.486**	-0.175	.385*	-0.207
Al	.368*	0.024	0.335	-.485**	.532**	-0.32
Cr	0.295	-0.028	-0.248	-0.351	0.303	-0.321
Pb	0.185	-0.128	0.255	-0.194	.439*	0.171
Syringic acid	0.153	0.195	.585**	0.208	.410*	0.147
Ferulic acid	-0.11	0.096	0.353	0.012	0.28	0.133
Chlorogenic acid	.472**	0.032	.811**	.503**	0.276	0.319
Caffeic acid	0.095	0.103	.550**	0.086	.520**	0.107
Gallic acid	.376*	0.013	.575**	.440*	0.23	0.299
Phthalic acid	0.037	-0.072	.390*	-0.111	.677**	0.073
Protocatechuic acid	0.296	0.333	.633**	0.317	0.359	0.054
p-Coumaric acid	0.011	0.192	0.116	0.186	-0.039	0.087
o-Coumaric acid	0.31	.454*	-0.186	0.338	-.400*	-0.286
Catechol	0.155	0	.618**	0.299	.403*	0.308
Benzoic acid	0.117	0.016	0.352	-0.031	.622**	0.036
p-Hydroxybenzoic acid	.518**	0.018	.771**	.417*	.379*	0.263
Protocatechuic aldehyde	.420*	0.074	.736**	.470**	0.348	0.27
Vanillin	-0.023	0.217	.387*	0.218	0.189	0.197
Sinapic acid	0.141	0.143	.510**	-0.098	.536**	-0.099
Kaempferol	0.264	0.128	.620**	.438*	0.325	0.348
Epicatechin	0.006	0.029	-0.163	0.359	-0.329	0.33
Catechin	0.239	0.212	-.383*	0.344	-0.346	-0.184
Chlorogenic acid	0.128	.539**	-0.064	.539**	-0.206	0.047
Rutin	.647**	0.223	0.083	-0.179	-0.038	-.586**
Vanillic acid	0.166	0.246	.588**	0.284	0.349	0.196
Oleanolic acid	-0.029	0.264	0.012	.471**	-0.244	0.118
Vanillin	-0.344	0.126	-.377*	0.288	-.585**	0.281
Glucosamine	0.114	-.392*	0.07	0.101	0.036	0.164
Mannosamine	-0.282	0.343	-.706**	0.284	-.496**	0.017
Galactosamine	-.453*	-0.135	-.612**	-.403*	0.019	-0.009
	**Alkaline protease**	**As**	**Cd**	**Al**	**Cr**	**Pb**
Morel yield	-.462*	-.561**	-0.188	-.477**	-0.049	0.067
pH	0.357	0.165	0.205	0.028	0.003	-0.165
Electrical conductivity	0.14	.549**	.513**	0.28	-0.143	0.041
Organic matter	-0.183	-0.258	-0.146	0.015	0.224	0.36
Ammonium N	-0.022	.750**	.782**	.548**	0.176	0.185
Nitrate N	-0.151	-0.213	-0.329	0.225	.399*	0.321

Available P	-.408*	-0.34	-0.106	-0.238	0.193	0.023
Available K	.415*	.500**	0.328	.368*	0.295	0.185
Urease	0.102	0.097	0.209	0.024	-0.028	-0.128
Sucrase	.419*	.642**	.486**	0.335	-0.248	0.255
Alkaline phosphatase	0.261	-0.296	-0.175	-.485**	-0.351	-0.194
Catalase	0.191	.620**	.385*	.532**	0.303	.439*
Polyphenol oxidase	0.021	-0.292	-0.207	-0.32	-0.321	0.171
Alkaline protease	1	.437*	0.179	0.22	-0.277	0.011
As	.437*	1	.820**	.831**	0.223	0.306
Cd	0.179	.820**	1	.695**	0.293	0.238
Al	0.22	.831**	.695**	1	.566**	.554**
Cr	-0.277	0.223	0.293	.566**	1	.486**
Pb	0.011	0.306	0.238	.554**	.486**	1
Syringic acid	0.284	.379*	0.359	-0.012	-0.351	0.048
Ferulic acid	-0.045	0.148	0.255	-0.083	-0.223	0.209
Chlorogenic acid	0.282	.386*	0.34	-0.021	-0.309	-0.083
Caffeic acid	0.352	.439*	.406*	0.05	-0.315	0.013
Gallic acid	-0.138	0.201	0.311	-0.098	-0.061	-0.087
Phthalic acid	0.357	.361*	0.215	0.078	-0.179	0.105
Protocatechuic acid	.514**	.561**	.499**	0.156	-0.342	-0.033
p-Coumaric acid	-0.078	-0.171	-0.084	-0.282	-0.17	0.276
o-Coumaric acid	0.314	0.12	0.135	0.267	0.226	0.018
Catechol	0.252	.367*	.504**	-0.059	-0.273	-0.146
Benzoic acid	.383*	0.31	0.138	0.007	-0.205	0.059
p-Hydroxybenzoic acid	0.217	.383*	0.324	-0.022	-0.227	-0.059
Protocatechuic aldehyde	.375*	0.342	0.302	-0.086	-0.323	-0.011
Vanillin	0.095	0.105	0.165	-0.181	-.364*	0.141
Sinapic acid	0.336	.535**	.464**	0.229	-0.179	0.169
Kaempferol	0.258	0.186	0.153	-0.235	-.424*	-0.009
Epicatechin	0.292	-0.34	-0.232	-.409*	-0.279	-0.325
Catechin	0.273	-0.265	-0.307	-0.054	0.27	0.158
Chlorogenic acid	0.227	-0.152	-0.347	-0.185	-0.261	0.02
Rutin	0.266	0.3	0.094	.393*	0.302	.371*
Vanillic acid	0.229	0.32	0.3	-0.08	-.404*	0.026
Oleanolic acid	0.335	0.001	-0.086	0.02	-0.185	-0.07
Vanillin	-.383*	-.688**	-.482**	-.483**	-0.094	0.211
Glucosamine	-0.236	-0.106	0.225	-0.09	.388*	0.08
Mannosamine	-.456*	-.554**	-0.094	-.392*	0.262	-0.178
Galactosamine	-0.285	-0.321	-0.208	0.054	.440*	.421*
	**Syringic acid**	**Ferulic acid**	**Chlorogenic acid**	**Caffeic acid**	**Gallic acid**	**Phthalic acid**
Morel yield	-0.151	0.116	0.061	-0.274	0.269	-.431*
pH	0.35	0.095	0.319	.467**	0.278	.513**
Electrical conductivity	.742**	.638**	.375*	.843**	0.283	.830**
Organic matter	-.384*	-0.191	-0.143	-.540**	-0.077	-.653**
Ammonium N	.581**	.545**	0.336	.643**	0.339	.565**
Nitrate N	-.711**	-.474**	-.799**	-.736**	-.761**	-.569**
Available P	-0.178	-0.086	0.076	-.366*	0.298	-.551**
Available K	0.153	-0.11	.472**	0.095	.376*	0.037
Urease	0.195	0.096	0.032	0.103	0.013	-0.072
Sucrase	.585**	0.353	.811**	.550**	.575**	.390*
Alkaline phosphatase	0.208	0.012	.503**	0.086	.440*	-0.111
Catalase	.410*	0.28	0.276	.520**	0.23	.677**
Polyphenol oxidase	0.147	0.133	0.319	0.107	0.299	0.073
Alkaline protease	0.284	-0.045	0.282	0.352	-0.138	0.357
As	.379*	0.148	.386*	.439*	0.201	.361*
Cd	0.359	0.255	0.34	.406*	0.311	0.215
Al	-0.012	-0.083	-0.021	0.05	-0.098	0.078
Cr	-0.351	-0.223	-0.309	-0.315	-0.061	-0.179
Pb	0.048	0.209	-0.083	0.013	-0.087	0.105
Syringic acid	1	.865**	.656**	.972**	.498**	.843**
Ferulic acid	.865**	1	0.33	.816**	0.29	.719**
Chlorogenic acid	.656**	0.33	1	.589**	.887**	.372*
Caffeic acid	.972**	.816**	.589**	1	.418*	.918**
Gallic acid	.498**	0.29	.887**	.418*	1	0.212
Phthalic acid	.843**	.719**	.372*	.918**	0.212	1
Protocatechuic acid	.901**	.615**	.724**	.892**	.486**	.712**
p-Coumaric acid	.617**	.845**	0.125	.503**	0.106	.454*
o-Coumaric acid	-0.356	-.469**	-0.118	-.389*	-0.202	-.494**
Catechol	.815**	.598**	.789**	.840**	.722**	.664**
Benzoic acid	.844**	.700**	.382*	.902**	0.215	.985**
p-Hydroxybenzoic acid	.685**	.376*	.973**	.628**	.905**	.461*
Protocatechuic aldehyde	.867**	.611**	.908**	.816**	.758**	.654**
Vanillin	.909**	.962**	.427*	.837**	0.324	.721**
Sinapic acid	.926**	.847**	.429*	.948**	0.246	.892**
Kaempferol	.910**	.733**	.786**	.847**	.660**	.720**
Epicatechin	-0.279	-.369*	-0.098	-0.242	-0.165	-0.234
Catechin	-0.306	-0.27	-0.285	-0.341	-0.331	-0.262
Chlorogenic acid	0.251	0.115	0.155	0.139	0.014	0.109
Rutin	-0.095	-0.036	-0.172	-0.155	-0.3	-0.111
Vanillic acid	.986**	.846**	.695**	.932**	.553**	.783**
Oleanolic acid	-0.004	-0.192	0.156	-0.041	-0.005	-0.136
Vanillin	-0.14	0.239	-0.355	-0.284	-0.244	-0.314
Glucosamine	-0.062	0.062	0.186	-0.05	.419*	-0.103
Mannosamine	-0.256	-0.025	-.376*	-0.28	-0.066	-0.336
Galactosamine	-0.314	0.084	-.803**	-0.261	-.654**	-0.036
	**Protocatechuic acid**	**p-Coumaric acid**	**o-Coumaric acid**	**Catechol**	**Benzoic acid**	**p-Hydroxybenzoic acid**
Morel yield	-0.317	0.308	-0.06	0.03	-.446*	0.004
pH	.455*	-0.196	-0.009	.582**	.481**	0.319
Electrical conductivity	.672**	0.16	-.504**	.708**	.751**	.422*
Organic matter	-0.316	0.155	.613**	-.483**	-.636**	-0.258

Ammonium N	.514**	0.135	-.397*	.581**	.495**	.413*
Nitrate N	-.677**	-0.127	.421*	-.954**	-.542**	-.807**
Available P	-0.169	0.222	.404*	-0.204	-.472**	0.054
Available K	0.296	0.011	0.31	0.155	0.117	.518**
Urease	0.333	0.192	.454*	0	0.016	0.018
Sucrase	.633**	0.116	-0.186	.618**	0.352	.771**
Alkaline phosphatase	0.317	0.186	0.338	0.299	-0.031	.417*
Catalase	0.359	-0.039	-.400*	.403*	.622**	.379*
Polyphenol oxidase	0.054	0.087	-0.286	0.308	0.036	0.263
Alkaline protease	.514**	-0.078	0.314	0.252	.383*	0.217
As	.561**	-0.171	0.12	.367*	0.31	.383*
Cd	.499**	-0.084	0.135	.504**	0.138	0.324
Al	0.156	-0.282	0.267	-0.059	0.007	-0.022
Cr	-0.342	-0.17	0.226	-0.273	-0.205	-0.227
Pb	-0.033	0.276	0.018	-0.146	0.059	-0.059
Syringic acid	.901**	.617**	-0.356	.815**	.844**	.685**
Ferulic acid	.615**	.845**	-.469**	.598**	.700**	.376*
Chlorogenic acid	.724**	0.125	-0.118	.789**	.382*	.973**
Caffeic acid	.892**	.503**	-.389*	.840**	.902**	.628**
Gallic acid	.486**	0.106	-0.202	.722**	0.215	.905**
Phthalic acid	.712**	.454*	-.494**	.664**	.985**	.461*
Protocatechuic acid	1	0.325	0.002	.786**	.713**	.691**
p-Coumaric acid	0.325	1	-0.316	0.254	.493**	0.179
o-Coumaric acid	0.002	-0.316	1	-0.35	-.468**	-0.263
Catechol	.786**	0.254	-0.35	1	.635**	.805**
Benzoic acid	.713**	.493**	-.468**	.635**	1	.481**
p-Hydroxybenzoic acid	.691**	0.179	-0.263	.805**	.481**	1
Protocatechuic aldehyde	.846**	.437*	-0.218	.867**	.674**	.915**
Vanillin	.698**	.865**	-.399*	.619**	.730**	.459*
Sinapic acid	.840**	.576**	-0.305	.671**	.873**	.474**
Kaempferol	.777**	.596**	-.438*	.819**	.755**	.832**
Epicatechin	-0.273	-0.209	-0.088	0.001	-0.182	-0.089
Catechin	-0.162	0.092	.732**	-.431*	-0.203	-0.35
Chlorogenic acid	.365*	0.281	.421*	-0.117	0.189	0.089
Rutin	-0.079	0.261	0.295	-.389*	-0.045	-0.131
Vanillic acid	.879**	.626**	-.371*	.794**	.797**	.724**
Oleanolic acid	0.303	-0.195	.732**	-0.08	-0.137	-0.022
Vanillin	-0.36	.608**	0.022	-.366*	-0.283	-.388*
Glucosamine	-0.158	0.071	-0.069	0.324	-0.145	0.209
Mannosamine	-0.322	0.137	0.209	-0.104	-0.317	-.368*
Galactosamine	-.510**	0.243	-0.074	-.467**	-0.07	-.746**
	**Protocatechuic aldehyde**	**Vanillin**	**Sinapic acid**	**Kaempferol**	**Epicatechin**	**Catechin**
Morel yield	0.007	0.103	-.364*	0.038	0.203	0.08
pH	.399*	0.114	0.295	0.308	0.091	0.001
Electrical conductivity	.509**	.553**	.815**	.528**	-.362*	-.599**
Organic matter	-0.235	-0.163	-.433*	-0.324	-0.165	.617**
Ammonium N	.393*	.408*	.705**	.369*	-.407*	-.625**
Nitrate N	-.813**	-.504**	-.517**	-.769**	-0.049	.504**
Available P	-0.026	-0.017	-.365*	-0.026	-0.017	0.353
Available K	.420*	-0.023	0.141	0.264	0.006	0.239
Urease	0.074	0.217	0.143	0.128	0.029	0.212
Sucrase	.736**	.387*	.510**	.620**	-0.163	-.383*
Alkaline phosphatase	.470**	0.218	-0.098	.438*	0.359	0.344
Catalase	0.348	0.189	.536**	0.325	-0.329	-0.346
Polyphenol oxidase	0.27	0.197	-0.099	0.348	0.33	-0.184
Alkaline protease	.375*	0.095	0.336	0.258	0.292	0.273
As	0.342	0.105	.535**	0.186	-0.34	-0.265
Cd	0.302	0.165	.464**	0.153	-0.232	-0.307
Al	-0.086	-0.181	0.229	-0.235	-.409*	-0.054
Cr	-0.323	-.364*	-0.179	-.424*	-0.279	0.27
Pb	-0.011	0.141	0.169	-0.009	-0.325	0.158
Syringic acid	.867**	.909**	.926**	.910**	-0.279	-0.306
Ferulic acid	.611**	.962**	.847**	.733**	-.369*	-0.27
Chlorogenic acid	.908**	.427*	.429*	.786**	-0.098	-0.285
Caffeic acid	.816**	.837**	.948**	.847**	-0.242	-0.341
Gallic acid	.758**	0.324	0.246	.660**	-0.165	-0.331
Phthalic acid	.654**	.721**	.892**	.720**	-0.234	-0.262
Protocatechuic acid	.846**	.698**	.840**	.777**	-0.273	-0.162
p-Coumaric acid	.437*	.865**	.576**	.596**	-0.209	0.092
o-Coumaric acid	-0.218	-.399*	-0.305	-.438*	-0.088	.732**
Catechol	.867**	.619**	.671**	.819**	0.001	-.431*
Benzoic acid	.674**	.730**	.873**	.755**	-0.182	-0.203
p-Hydroxybenzoic acid	.915**	.459*	.474**	.832**	-0.089	-0.35
Protocatechuic aldehyde	1	.704**	.694**	.935**	-0.129	-0.176
Vanillin	.704**	1	.835**	.833**	-0.256	-0.194
Sinapic acid	.694**	.835**	1	.720**	-.408*	-0.246
Kaempferol	.935**	.833**	.720**	1	-0.037	-0.302
Epicatechin	-0.129	-0.256	-.408*	-0.037	1	-0.039
Catechin	-0.176	-0.194	-0.246	-0.302	-0.039	1
Chlorogenic acid	0.227	0.29	0.152	0.249	-0.28	.495**
Rutin	-0.087	-0.02	0.085	-0.131	-0.12	.400*
Vanillic acid	.873**	.910**	.870**	.939**	-0.252	-0.336
Oleanolic acid	0.087	-0.08	-0.061	-0.083	-0.274	.544**
Vanillin	-0.228	0.261	-0.23	-0.063	0.057	0.359
Glucosamine	0.185	-0.042	-0.12	0.044	0.089	0.101
Mannosamine	-0.3	-0.058	-0.325	-0.242	0.217	0.319
Galactosamine	-.583**	-0.042	-0.119	-.441*	-0.027	0.293
	**Chlorogenic acid**	**Rutin**	**Vanillic acid**	**Oleanolic acid**	**Vanillin**	**Glucosamine**
Morel yield	-0.152	-0.236	-0.1	-0.068	.714**	.600**
pH	0.043	-.559**	0.285	0.27	-.447*	0.195

Electrical conductivity	-0.174	-0.342	.679**	-0.156	-.502**	-0.069
Organic matter	0.326	0.353	-0.341	.412*	.649**	0.168
Ammonium N	-.415*	0.027	.524**	-.415*	-.516**	0.103
Nitrate N	0.134	.544**	-.707**	0.061	.386*	-0.309
Available P	.362*	0.186	-0.082	0.169	.533**	0.173
Available K	0.128	.647**	0.166	-0.029	-0.344	0.114
Urease	.539**	0.223	0.246	0.264	0.126	-.392*
Sucrase	-0.064	0.083	.588**	0.012	-.377*	0.07
Alkaline phosphatase	.539**	-0.179	0.284	.471**	0.288	0.101
Catalase	-0.206	-0.038	0.349	-0.244	-.585**	0.036
Polyphenol oxidase	0.047	-.586**	0.196	0.118	0.281	0.164
Alkaline protease	0.227	0.266	0.229	0.335	-.383*	-0.236
As	-0.152	0.3	0.32	0.001	-.688**	-0.106
Cd	-0.347	0.094	0.3	-0.086	-.482**	0.225
Al	-0.185	.393*	-0.08	0.02	-.483**	-0.09
Cr	-0.261	0.302	-.404*	-0.185	-0.094	.388*
Pb	0.02	.371*	0.026	-0.07	0.211	0.08
Syringic acid	0.251	-0.095	.986**	-0.004	-0.14	-0.062
Ferulic acid	0.115	-0.036	.846**	-0.192	0.239	0.062
Chlorogenic acid	0.155	-0.172	.695**	0.156	-0.355	0.186
Caffeic acid	0.139	-0.155	.932**	-0.041	-0.284	-0.05
Gallic acid	0.014	-0.3	.553**	-0.005	-0.244	.419*
Phthalic acid	0.109	-0.111	.783**	-0.136	-0.314	-0.103
Protocatechuic acid	.365*	-0.079	.879**	0.303	-0.36	-0.158
p-Coumaric acid	0.281	0.261	.626**	-0.195	.608**	0.071
o-Coumaric acid	.421*	0.295	-.371*	.732**	0.022	-0.069
Catechol	-0.117	-.389*	.794**	-0.08	-.366*	0.324
Benzoic acid	0.189	-0.045	.797**	-0.137	-0.283	-0.145
p-Hydroxybenzoic acid	0.089	-0.131	.724**	-0.022	-.388*	0.209
Protocatechuic aldehyde	0.227	-0.087	.873**	0.087	-0.228	0.185
Vanillin	0.29	-0.02	.910**	-0.08	0.261	-0.042
Sinapic acid	0.152	0.085	.870**	-0.061	-0.23	-0.12
Kaempferol	0.249	-0.131	.939**	-0.083	-0.063	0.044
Epicatechin	-0.28	-0.12	-0.252	-0.274	0.057	0.089
Catechin	.495**	.400*	-0.336	.544**	0.359	0.101
Chlorogenic acid	1	0.084	0.309	.718**	0.265	-.544**
Rutin	0.084	1	-0.112	-0.168	0.095	-0.116
Vanillic acid	0.309	-0.112	1	0	-0.09	-0.108
Oleanolic acid	.718**	-0.168	0	1	0.025	-0.271
Vanillin	0.265	0.095	-0.09	0.025	1	0.085
Glucosamine	-.544**	-0.116	-0.108	-0.271	0.085	1
Mannosamine	-0.038	-0.248	-0.252	-0.014	.501**	.421*
Galactosamine	-0.158	0.094	-.371*	-0.229	.506**	0.107

	**Mannosamine**	**Galactosamine**
Morel yield	.526**	0.152
pH	0.096	-0.081
Electrical conductivity	-0.357	-0.13
Organic matter	0.281	0.162
Ammonium N	-0.303	-0.141
Nitrate N	0.126	.567**
Available P	.548**	-0.079
Available K	-0.282	-.453*
Urease	0.343	-0.135
Sucrase	-.706**	-.612**
Alkaline phosphatase	0.284	-.403*
Catalase	-.496**	0.019
Polyphenol oxidase	0.017	-0.009
Alkaline protease	-.456*	-0.285
As	-.554**	-0.321
Cd	-0.094	-0.208
Al	-.392*	0.054
Cr	0.262	.440*
Pb	-0.178	.421*
Syringic acid	-0.256	-0.314
Ferulic acid	-0.025	0.084
Chlorogenic acid	-.376*	-.803**
Caffeic acid	-0.28	-0.261
Gallic acid	-0.066	-.654**
Phthalic acid	-0.336	-0.036
Protocatechuic acid	-0.322	-.510**
p-Coumaric acid	0.137	0.243
o-Coumaric acid	0.209	-0.074
Catechol	-0.104	-.467**
Benzoic acid	-0.317	-0.07
p-Hydroxybenzoic acid	-.368*	-.746**
Protocatechuic aldehyde	-0.3	-.583**
Vanillin	-0.058	-0.042
Sinapic acid	-0.325	-0.119
Kaempferol	-0.242	-.441*
Epicatechin	0.217	-0.027
Catechin	0.319	0.293
Chlorogenic acid	-0.038	-0.158
Rutin	-0.248	0.094
Vanillic acid	-0.252	-.371*
Oleanolic acid	-0.014	-0.229
Vanillin	.501**	.506**
Glucosamine	.421*	0.107
Mannosamine	1	.456*
Galactosamine	.456*	1

*$p < 0.05$, **$p < 0.01$, ***$p < 0.001$. For the Pearson correlation analysis, a total of 30 soil samples were used, and the analysis integrated data collected from both the 2024 and 2025 growing seasons.
